# Structural and Functional Analysis of Gly212 Mutants Reveals the Importance of Intersubunit Interactions in ASIC1a Channel Function

**DOI:** 10.3389/fmolb.2020.00058

**Published:** 2020-04-28

**Authors:** Olivier Bignucolo, Sabrina Vullo, Nicolas Ambrosio, Ivan Gautschi, Stephan Kellenberger

**Affiliations:** ^1^Department of Biomedical Sciences, University of Lausanne, Lausanne, Switzerland; ^2^SIB Swiss Institute of Bioinformatics, Lausanne, Switzerland

**Keywords:** ion channel, current kinetics, subunit interaction, structure-function relationship, salt bridge, molecular dynamics

## Abstract

Acid-sensing ion channels (ASICs) act as pH sensors in neurons. ASICs contribute to pain sensation, learning, fear behavior and to neuronal death after ischemic stroke. Extracellular acidification induces a transient activation and subsequent desensitization of these Na^+^-selective channels. ASICs are trimeric channels made of identical or homologous subunits. We have previously shown that mutation of the highly conserved Gly212 residue of human ASIC1a to Asp affects the channel function. Gly212 is located in the proximity of a predicted Cl^–^ binding site at a subunit interface. Here, we have measured the function of a series of Gly212 mutants. We show that substitution of Gly212 affects the ASIC1a pH dependence and current decay kinetics. Intriguingly, the mutations to the acidic residues Asp and Glu have opposing effects on the pH dependence and the current decay kinetics. Analysis of molecular dynamics simulation trajectories started with the coordinates of the closed conformation indicates that the immediate environment of residue 212 in G212E, which shifts the pH dependence to more alkaline values, adopts a conformation closer to the open state. The G212D and G212E mutants have a different pattern of intersubunit salt bridges, that, in the case of G212E, leads to an approaching of neighboring subunits. Based on the comparison of crystal structures, the conformational changes in this zone appear to be smaller during the open-desensitized transition. Nevertheless, MD simulations highlight differences between mutants, suggesting that the changed function upon substitution of residue 212 is due to differences in intra- and intersubunit interactions in its proximity.

## Introduction

**FIGURE 1:**
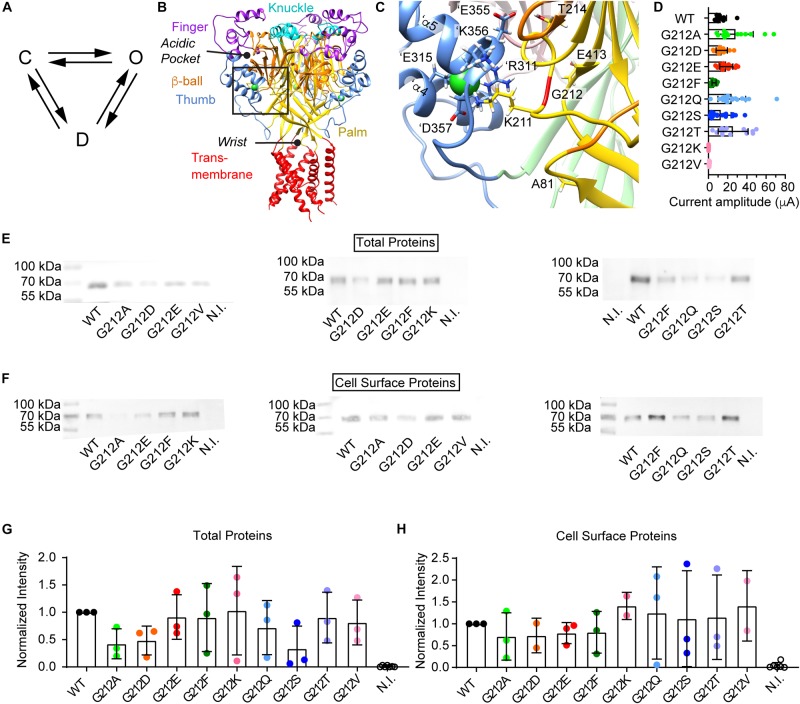
Current and protein expression of Gly212 mutants. **(A)** Kinetic scheme of ASIC1a activity; C, closed state; O, open state; D, desensitized state. **(B–C)** The structural images were obtained from a model of human ASIC1a based on the crystal structure of MitTx-opened chicken ASIC1a (4NTW, Baconguis et al., 2014). **(B)** Trimeric ASIC1a, with domains colored as indicated. The position of the acidic pocket and the wrist are highlighted. The predicted positions of the Cl^–^ ions are indicated by green spheres. The black frame indicates the area of the zoom shown in **(C)**. **(C)** Environment of Gly212, with indication of important residues. Gly212 is marked in red. The same color code as in **(B)** is used, except for the β-ball and palm of the subunit adjacent to the one of which Gly212 is shown. In this neighboring subunit, the β-ball is pink, and the palm is light green. Residue labels of the neighboring subunit are preceded by the prefix ‘. **(D)** Dot-plot of maximal current amplitudes of WT and the Gly212 mutants. Conditioning pH7.4 (7.8 for G212F) was applied for 1 min followed by a 10 s stimulation step to pH pH4.5 for the WT and the mutants G212A, -D, -E -F, -Q, -S and -T, and pH4.0 for the mutants G212K and -V (*n* = 7–21). For electrophysiology experiments, *Xenopus laevis* oocytes were injected with 1–44 ng RNA per oocyte (WT: 2.5, G212D:1, G212E: 5, G212T: 5, G212V, -A, and -Q: 24, G212K: 32 and G212F: 44 ng). **(E–H)** For the biochemistry analysis, the same amount of cRNA, 10 ng per oocyte, was injected for all mutants. **(E)** Western blot of total proteins. Anti-ASIC1 antibody was used to detect the proteins. **(F)** Western blot images of biotinylated cell surface proteins. Anti-ASIC1 antibody was used to detect the proteins. **(G)** Total protein quantification. Gel background intensity is subtracted from the band intensity and values are normalized to the WT signal on the same blot. **(H)** Biotinylated surface protein quantification. Gel background intensity is subtracted from the band intensity and values are normalized to the WT. N.I., non-injected.

Acid-sensing ion channels (ASICs) are Na^+^-selective ion channels that form a subgroup within the Epithelial Na^+^ Channel/Degenerin family of ion channels (Eastwood and Goodman, 2012; Kellenberger and Schild, 2015). ASICs are activated by extracellular acidification. The six main subunits in rodents are ASIC1a, ASIC1b, ASIC2a, ASIC2b, ASIC3, and ASIC4. ASIC1a, -2a, -2b, and -4 are expressed in the CNS, and all ASIC subunits except ASIC4 are expressed in the PNS (Wemmie et al., 2013; Kellenberger and Schild, 2015). So far, no channel function has been found for ASIC4, and it has been shown that ASIC2b cannot form functional homotrimeric channels (Lingueglia et al., 1997; Lin et al., 2015). ASIC2b can, however, participate in the formation of functional heterotrimeric ASIC channels, and ASIC1a, -1b, -2a, and -3 can form homo- and heterotrimeric channels. Studies with ASIC subtype-specific knockout mice and with the use of specific inhibitors demonstrated roles of ASICs in fear behavior, learning, neurodegeneration after ischemic stroke and in pain sensation (Price et al., 2001; Wemmie et al., 2002; Xiong et al., 2004; Deval et al., 2008; Ziemann et al., 2009). Extracellular acidification induces an ASIC current that is transient, because it is terminated by the transition to a non-conducting state called desensitization. ASICs can therefore exist in three functional states: closed, open and desensitized (Wemmie et al., 2013; Grunder and Pusch, 2015; Kellenberger and Schild, 2015; Figure 1A). Crystal structures of chicken ASIC1a, which shares 90% sequence homology with human ASIC1a, have been published for the desensitized, closed, and toxin-opened conformations (Jasti et al., 2007; Gonzales et al., 2009; Baconguis and Gouaux, 2012; Dawson et al., 2012; Baconguis et al., 2014; Yoder et al., 2018). The intracellular *N-* and *C-*termini were not resolved in these structures. The topology of the single ASIC subunit was compared to the shape of a hand holding a small ball, with the two transmembrane domains corresponding to the forearm. Sub-domains were in accordance labeled as palm, finger, knuckle, thumb, and β-ball (Figure 1B). Three subunits are arranged around a central vertical axis to form a functional channel, with the finger and thumb domains turned away from the central axis, toward the extracellular space. In each subunit, the finger, thumb, and β-ball enclose a cavity called the “acidic pocket” because of the high density of acidic residues. The area just above the extracellular pore entry was labeled as “wrist.” Comparison of the structures representing different functional states indicates during the closed-open transition a collapse of the acidic pocket and the opening of the wrist and the pore (Yoder et al., 2018). There is also a general increase in intersubunit distances. Upon desensitization, the lower palm domains collapse around the central cavity, and the channel pore closes (Roy et al., 2013; Baconguis et al., 2014).

Based on the structural information it was proposed that the acidification-induced collapse of the acidic pocket leads to pore opening. Several studies have, however, shown that the ASIC1a pH dependence is controlled by additional protonation sites in the palm and the wrist (Paukert et al., 2008; Liechti et al., 2010; Krauson et al., 2013). Interestingly, channel mutants in which all the acidic residues in the acidic pocket region had been mutated exhibited normal acidification-activated currents (Vullo et al., 2017). These findings indicate that pore opening is not driven by the collapse of the acidic pocket alone. Changes in intersubunit contacts are known to occur during ASIC activity (Gwiazda et al., 2015; Yoder et al., 2018). In an analysis of the evolution of ASICs, Lys211, located at a subunit interface close to the acidic pocket, was proposed as a determinant of proton sensitivity (Lynagh et al., 2018). We have recently shown that the human ASIC1a WT clone that we and many other groups have used, contained a mutation of the adjacent residue, Gly212 to Asp. This mutation slightly changed the pH dependence, and accelerated the decay kinetics of ASIC1a currents (Vaithia et al., 2019). Gly212 is located close to a Cl^–^ binding site that was predicted in the open and desensitized, but not closed ASIC1a (Yoder and Gouaux, 2018; Figure 1C). The residues presented in Figure 1C belong to two subunits, one subunit on the left (containing the suffix’ in the labels), where residues of mostly the thumb are marked, and the other subunit on the right, showing labeled residues of the palm. The ASIC1a current desensitization kinetics are strongly influenced by the type of anion in the solution (Kusama et al., 2010, 2013). To understand the role of this channel area in ASIC1a activation and desensitization, we have replaced in the present study Gly212 by several other residues, and have measured the pH dependence and current kinetics of these mutants. Mutation of Gly212 to Glu shifted the pH dependence of activation to more alkaline values, while most of the other tested mutations induced an acidic shift. The current decay kinetics were strongly slowed in the mutants G212E and G212A, and accelerated in G212F. In all mutants, the current decay kinetics depended on the Cl^–^ concentration. The analysis of molecular dynamics (MD) simulation trajectories of WT ASIC1a and selected mutants identified differences in intra- and intersubunit interactions between mutants that likely underlie the observed differences in current properties. Specifically, trajectories conducted with Glu at position 212, started from the closed state, adopted within a few hundreds of nanoseconds several features characteristic of the open crystal structure, whereas with Asp or Phe at position 212 the protein did not depart from the closed conformation within this simulation time.

## Materials and Methods

### Molecular Biology

The human ASIC1a sequence was subcloned into a pSP65-derived vector containing 5′ and 3′ non-translated sequences of β globin to improve the stability and the expression in *Xenopus laevis* oocytes. Amino acid substitutions were generated by site-directed mutagenesis using KAPA HiFi HotStart PCR polymerase (KAPA Biosystems), using the Quikchange approach. Mutations were verified by sequencing (Microsynth). *In vitro* transcription was performed using the mMESSAGE mMACHINE SP6 kit (Ambion/Life Technologies).

### Oocyte Handling and Injection

All experiments with *Xenopus laevis* oocytes were carried out in accordance with the Swiss federal law on animal welfare and had been approved by the committee on animal experimentation of the Canton de Vaud. After surgical removal, healthy stage V and VI oocytes of female *Xenopus* frogs were treated with collagenase for isolation and defolliculation. They were subsequently injected with 50 nl (0.02–0.9 μg/μl) of cRNA. After injection, they were kept at 19°C in Modified Barth’s Solution (MBS) composed of (mM): 85 NaCl, 1 KCl, 2.4 NaHCO_3_, 0.33 Ca(NO_3_)_2_, 0.82 MgSO_4_, 0.41 CaCl_2_, 10 HEPES and 4.08 NaOH. Experiments were performed 24–48 h after injection.

### Electrophysiological Measurements From *Xenopus* Oocytes

Standard recording solutions contained (mM) 110 NaCl, 2 CaCl_2_, and 10 HEPES for pH ≥ 6.8. For solutions with a pH < 6.8, HEPES was replaced by 10 mM MES. The pH was adjusted using NaOH. For some experiments, Cl^–^ was completely replaced by the same concentration of SCN^–^. Whole-cell currents from *Xenopus* oocytes were recorded by two-electrode voltage clamp (TEV-200A; Dagan Corporation) at -40 mV using Chartmaster software (HEKA Electronics) at a sampling rate of 1 ms and low-pass filtering at 2 kHz. Oocytes were placed in a RC*-*26Z recording chamber (Warner Instruments) and impaled with two glass electrodes filled with 1 M KCl, with a resistance of <0.5 MΩ. Oocytes were perfused at a rate of 5–15 ml/min. All experiments were performed at room temperature (20–25°C).

### Electrophysiology Data Analysis and Statistics

Data were analyzed with the software FitMaster (HEKA Electronics). pH response curves for H^+^ activation were fitted with a Hill function: I = I_max_/(1+ (10^–pH50^/10^–pH^)^nH^), where I_max_ is the maximal current, pH_50_ is the pH inducing 50% of the maximal current amplitude, and nH is the Hill coefficient. SSD curves were fitted with an analogous equation. Time constants of desensitization were determined by fitting the decay time of current traces to a mono-exponential function. The results are presented as mean ± SEM. They represent the mean of n independent experiments obtained from different cells. Statistical analysis was done with *t*-test where two conditions were compared, or with One-way ANOVA followed by Dunnett’s or Tukey multiple comparisons test (Graphpad Prism 6).

### Surface Protein Biotinylation and Western Blot

These experiments were carried out as described elsewhere (Jiang et al., 2010). Briefly, 20–30 healthy oocytes were transferred 24 h after injection to MBS solution. All procedures were performed on ice. Oocytes were exposed to 1 mg/ml of EZ-Link Sulfo-NHS-SS-Biotin (Thermo Fisher Scientific) in MBS for 15 min. The biotinylation reaction was quenched by adding 1.5 ml of quenching buffer (191 mM Glycine, 25 mM Tris HCL pH 7.5) for 5 min at RT, oocytes were then transferred in a new multiwell with MBS, and damaged oocytes were removed. Healthy oocytes were transferred in 1.5 ml tubes containing 1 ml of MBS. Oocyte lysates were prepared by incubation in lysis buffer containing 100 mM NaCl, 5 mM EDTA, 1% Triton X-100, 20 mM Tris HCL at pH7.5, supplemented with protease inhibitor cocktail (1 mM PMSF, 1 mg/ml Pepstatin, 10 mg/ml Aprotinin, 10 mg/ml Leupeptin). Oocytes were homogenized and centrifuged at 15,000 g at 4°C for 10 min. The supernatant was transferred to a new 1.5 ml tube. Samples for the determination of total ASIC protein expression were prepared by mixing 20 μl of the remaining supernatant with 20 μl of Lysis buffer and 10 μl of 5X sample loading buffer (1.5 M Sucrose, 10% SDS, 12.5 mM EDTA, 312 mM Tris pH8.8, 0.25% (w/v) bromophenol blue, 125 mM DTT), followed by heating at 95°C for 10 min. To determine surface ASIC protein expression, 50 μl of Streptavidin beads (Thermo Fisher Scientific) were added to each tube containing the lysed oocytes from the previous step, and put on a rotating wheel overnight at 4°C. Beads and supernatant were then separated by centrifugation at 2000 rpm for 3 min, the supernatant was discarded and the beads were washed 4 times with 1 ml of Lysis buffer without inhibitor for 3 min at 2000 rpm at 4°C. After washing, 50 μl of 2X sample loading buffer (20% glycerol, 6% SDS, 250 mM Tris-HCl at pH6.7, 0.1% (w/v) bromophenol blue, 50 mM DTT) was added to each sample, followed by heating at 95°C for 5 min and a short spin down. For both ASIC expression determinations, protein samples of 20 μl were separated on a 10% SDS-PAGE (running buffer: 27.5 mM Tris-base, 213 mM Glycine and 1% SDS) at 90 V for 15–20 min and then at 120 V for 1 h 20. Samples were transferred to Protran^TM^ 0.2 μM nitrocellulose membranes (Amersham Biosciences) at 4 °C, 120V for 1.5 h. After the transfer, the membranes were incubated 5 min in Ponceau red, rinsed, incubated 5 min in TBS + 1% Tween 20 (in mM, 137 NaCl, 2.7 KCl, 19 Tris base, 0.1% Tween 20) and blocked by TBST containing 5% BSA for 1 h. The protein samples on the membrane were exposed overnight at 4°C to anti-ASIC1 antibody (1:200, Alomone, Israel) in TBST buffer containing 1% BSA, washed 5 times, and were then exposed to Goat Anti-Rabbit IgG H&L horseradish peroxidase-linked secondary antibody (1:2000, Abcam, Switzerland) for 1 h at room temperature. The signals were detected using the Fusion SOLO chemiluminescence system (Vilber Lourmat, Marne-la-Vallée, France) using SuperSignal West Femto Maximum Sensitivity Substrate (Thermo Fisher Scientific). The band intensities were quantified by the linear analysis method of the software, with the area of measurement kept the same for all samples of the same blot. Background noise was subtracted prior to determining the intensity occupied by individual bands.

### Molecular Constructs for Computational Analysis

To date, no structure of human ASIC1a has been published. Since the chicken ASIC1 shares 90% identity with its human homolog, homology models were constructed on the basis of the chicken closed (PDB code 5WKU; Yoder et al., 2018) open (4NTW; Baconguis et al., 2014) and desensitized (4NYK; Gonzales et al., 2009; Baconguis et al., 2014) structures with SWISS-MODEL (Biasini et al., 2014). Using VMD (Humphrey et al., 1996) and Chimera (Pettersen et al., 2004) suites, extended conformations of *N-*termini, starting at residue 14, were created and grafted onto the open (but not other) hASIC1a structures. Supplementary Figure S1 shows an alignment of the complete human ASIC1a sequence and of the chicken and human ASIC1a sequences corresponding to the resolved crystal structure and homology model, respectively, of the closed state. Using the VMD Mutator plugin, the residue G212 was mutated into D, E, F, or T.

According to the crystal structures, the open and desensitized states do not differ much in the area around Gly212. For the simulations involving these two states, acidic residues were left unprotonated by default. Preliminary simulations with these states suggested that this setting sufficed for the desensitized state, whereas it was opted to perform pKa calculations for the open state. These calculations were performed using the PBEQ (Im et al., 1998) module of CHARMM (Brooks et al., 1983). The systems, as described above, were subjected to all-atom MD simulations for 10 ns, and coordinates of the protein and ions located within 3 Å of any residues, extracted at *t* = 1, 2.5, 5, and 10 ns were then submitted to the PBEQ module of CHARMM, and a calculation similar to the one performed by Liechti at al. was conducted (Liechti et al., 2010). The pKa was calculated as the mean of the values of each monomer obtained at the four time points. The total duration of 10 ns was chosen in order to be long enough to sample random diffusive motion around the titration candidate sites, without being so long to allow conformational drift. To limit differences in protonation between the simulations of the open and desensitized state, acidic residues were protonated if their pKa was shifted to values higher than 8. If side chains of two residues with pKa > 8 were within ∼5 Å from each other, only one of the two residues was protonated. The following residues were protonated: E97, E238, D351, and D409. The part of the analysis involving hASIC1a in the closed state aimed specifically at identifying early responses of a closed channel exposed to an acidic pH, so that in this case a more extensive protonation was applied. First, the pKas were estimated as above. With the aim of exposing the closed ASIC structure to acidic conditions (∼pH5) to induce channel opening, the residues having a calculated pKa > 5.3 were candidates for protonation. To decide on protonation or not of a given residue in the closed conformation, information from preliminary constant pH simulations was also considered (data not shown). Using the constant pH MD suite implemented in NAMD (Chen and Roux, 2015), simulations were conducted starting from the closed structure. Although the protonation in these preliminary simulations did not achieve convergence, the protonation state of titratable residues at pH 5.0 was assessed. This information was used to decide whether a given residue was protonated or not, if the calculated pKa of this residue was close to 5.3 (thus close to threshold). The following residues were protonated: E63, H70, H72, E97, H110, E113, H173, E177, E219, E238, E254, E321, D347, D351, E355, D409, E413, E418, D434, E452.

### Molecular Dynamics Simulations

Using the Charmm-gui server (Jo et al., 2009), the models were embedded in a phospholipid membrane. In the case of the simulations starting from the closed state, the membrane contained ∼125 1-palmitoyl-2-oleoyl-sn-glycero-3-phosphocholine (POPC) molecules in each leaflet. Simulations starting from the open/desensitized state were constructed with membranes containing POPC, 1,2-Dimyristoyl-sn-glycero-3-phosphoethanolamine, 1,2-Dimyristoyl-sn-glycero-3-phosphorylglycerol and 1,2-dioleoyl-sn-glycero-3-phospho-L-serine. It is unlikely that the lipid composition, which was different between simulations, would affect interactions in the proximity of Gly212, which is ∼40 Å distant from the plasma membrane. The NaCl concentration was set to 150 mM; the total number of ionic species deviates slightly from equality in order to ensure electrical neutrality of the complete systems (Supplementary Table S1).

All-atom MD simulations were performed with the CHARMM36 force field (Best et al., 2012), mostly with the GROMACS package, version 2016.3 (Van Der Spoel et al., 2005; Bjelkmar et al., 2010). The TIP3P water model was used (Jorgensen et al., 1983). Bond and angle lengths involving hydrogen atoms were constrained using the LINCS algorithm (Hess et al., 1997), allowing an integration time step of 2 fs. Short-range electrostatics were cut off at 1.2 nm. Van der Waals interactions were calculated explicitly up to a distance of 1.0 nm, beyond which a switch function was used to smoothly switch off the interactions to reach zero at 1.2 nm. Long-range electrostatic interactions were calculated by the PME algorithm (Essmann et al., 1995). The protein, lipids, and water/ions were coupled separately to a temperature bath with the Nose-Hoover method with a time constant of 1.0 ps (Nose, 1984; Hoover, 1985). The system pressure was kept constant by semiisotropic Parrinello-Rahman coupling to a reference value of 1 bar (Parrinello and Rahman, 1981; Nose and Klein, 1983). Some MD simulations were conducted with the NAMD 2.12 package (Phillips et al., 2005; Supplementary Table S2). In these trajectories, the SETTLE algorithm was used to constrain bond lengths and angles of water molecules, while the other hydrogens were constrained using the SHAKE algorithm. The electrostatic calculation parameters were the same as for the GROMACS calculations. The temperature of the system was kept constant by using Langevin dynamics with an external heat bath at 310 K. The anisotropic pressure was controlled using the Nose-Hoover method and standard parameters. The total simulated time was 5648 ns, and the simulations using the closed state exposed to acidic conditions accounted for 61% of the trajectories (Supplementary Table S2).

### Data Analysis of the Computational Part

Data were analyzed with R, python, VMD and tcl in-house scripts. When not stated otherwise, structures taken at 1 ns interval were analyzed and the first 20 ns of simulation were discarded, resulting in 180 frames per replication for structural analysis. The timeline plugin of VMD was used to extract RMSD values. The density function of R was used to fit the RMSD distributions and extract their maximal value. The orientation of the thumb α-helices in Figures 4H–J was determined as follows. The protein structures of all frames were aligned to the initial structure, oriented by CHARMM with its principal axis along the z-axis. Each α-helix was represented by a vector. The starting point of the vector was the center-of-mass (COM) of the backbone atoms of residues 307–310 and 353–356, respectively, for the α-helix 4 and 5. The ending point was formed by the COMs of the backbone atoms of residues 320–323 and 339–342, respectively. For each frame, the cosine of this vector in respect to the ideal z-axis was then calculated. The principal axis of the protein might deviate slightly from the ideal z-axis upon tumbling and conformational changes. Figures 4I,J suggests, however, that the large number of frames and replications compensated for this.

The local conformational changes were assessed through the investigation of the side-chain reorientation during the simulations. Torsion angles (χ1) were extracted and analyzed using in-house R and python scripts and the get-torsion script available at https://gist.github.com/lennax, which imports the PDB module from Biopython (Hamelryck and Manderick, 2003; Cock et al., 2009). In order to assess the global conformational changes, the approach introduced by Cunningham (2012) and Gaieb and Morikis (2017) was followed. In brief, using the change in the center-of-mass position of the Cα atoms between selected time intervals (mostly 1 ns, ensuring structural independency), a 40 × 40 cross-correlation matrix of the 40 residues was constructed, located within 10 Å of residue 212 in the crystal structure as follows:

(1) $$M_{i\,j}=\frac{1}{N}\,\sum \frac{\Delta \,x_{i}\cdot \Delta \,x_{j}}{\left|\Delta \,x_{i}\right|\cdot \left|\Delta \,x_{i}\right|}$$

where Δ represents the difference between two subsequent time frames, *i*, *j* stands for different Cα atoms, and the summation extends over all N frames in the trajectory. As suggested (Cunningham, 2012), residue pairs displaying highest absolute cross-correlations are expected to be involved in persistent interactions within the molecule. Since close neighbors are automatically involved in concerted motions, pairs involving residues separated by less than three residues in the sequence were removed from the heat map. On a heat map corresponding to a single trajectory, each residue pair is obviously represented only once, so that this analysis is not statistically robust against false positives. The sampling was thus increased in comparison to previous work by computation of a replicated heat map for each of the nine obtained trajectories. Additionally, subunit-independent motion was assumed, as supported by the relatively large distances between residues 212 of different subunits (>30 Å) and the ∼200 ns long simulations.

Accordingly, 27 heat maps were analyzed. The five pairs of residues with the highest positive cross-correlations were selected from the heat map, resulting in 45 pairs per construct. This first filter produces thus residue pairs with the highest cross-correlation values. From this dataset the 10 most frequently observed pairs were extracted, thus the most reproducible residue pairs for each of the three constructs (Supplementary Table S3). Thirdly, interacting residue clusters that were detected in all three constructs, thus G212E, G212D, and G212, were identified manually. This approach selects residues that show correlated motions independently of the mutation at position 212. Finally, pairs of interacting residues that are found among the best ranked only in a specific construct were selected, to identify clusters that were specific to G212E, G212D, or WT (G212).

## Results

### Unchanged Cell Surface Expression in Gly212 Mutants

Gly212 was mutated to the acidic residues Asp and Glu, to the basic residue Lys, to hydrophobic residues of different shapes and sizes (Ala, Phe, Val) and to the hydrophilic residues Ser, Thr and Gln. Mutant channels were expressed in *Xenopus laevis* oocytes, and currents were measured by two-electrode voltage-clamp. Most mutants expressed current amplitudes similar to that of WT ASIC1a (Figure 1D). Only G212V and G212K expressed very small current amplitudes. A Lys residue might not be tolerated at this position, because there are already two basic residues present, Lys211 and ’Arg311. The relatively large Val side chain might not be well accommodated in this hydrophilic environment. To determine whether the lower current expression in G212K and G212V was due to decreased channel expression or function, the total expression of the different mutants was determined by SDS-PAGE separation, western blot and labeling with an ASIC1-specific antibody. There were no significant differences in protein expression of any of the mutants compared to the WT (Figures 1E,G). ASICs expressed at the cell surface were then isolated by cell surface biotinylation. None of the Gly212 mutants showed a cell surface expression that was different from that of the WT (Figures 1F,H).

### Shifted pH Dependence of Activation and Steady-State Desensitization

**FIGURE 2:**
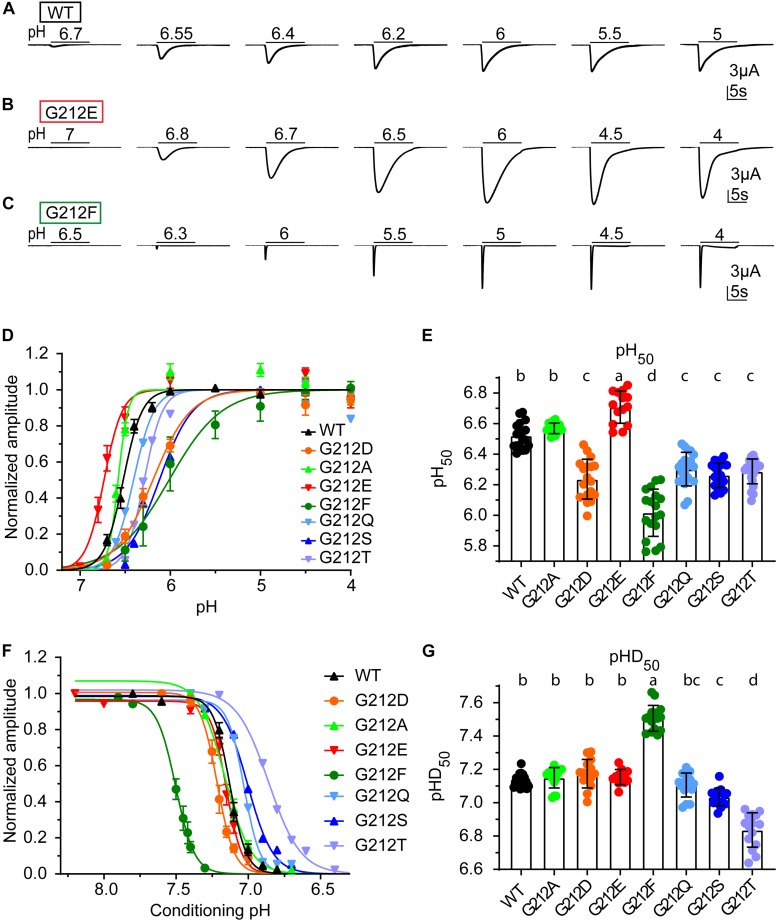
Functional differences of Gly212 mutants. **(A–C)** Representative current traces of activation curves with ASIC1a WT **(A)**, G212D **(B)** and G212F **(C)**. **(D)** pH dependence of activation of WT and Gly212 mutants (*n* = 13–22). A series of pH stimulations was applied for 10 s each, and between, oocytes were perfused with pH 7.4 (pH7.8 for G212F) conditioning solution during 50 s. **(E)** Plot of the pH_50_ (pH of half-maximal amplitude) values of WT and Gly212 mutants. Bars that do not share the same letters are significantly different (*n* = 14–22, *p* < 0.05). **(F)** Steady-state desensitization (SSD) curve, plotting the normalized current amplitude as a function of the conditioning pH, of WT and Gly212 mutants (*n* = 8–17). The curve was obtained by applying the conditioning pH for 55 s followed by application of pH5 for 5 s. **(G)** Plot of pHD_50_ values. Bars that do not share the same letters are significantly different (*n* = 12–21, *p* < 0.05).

The pH dependence of activation was determined by stimulating oocytes expressing human ASIC1a once every min during 10 s by a stimulation solution of different pH while the current was recorded. Representative current traces from such experiments are shown for WT (Figure 2A), G212E (Figure 2B) and G212F (Figure 2C). The normalized peak currents of such experiments are plotted as a function of the stimulation pH in Figure 2D for WT and mutant ASIC1a. From such experiments, the pH of half-maximal activation, pH_50_, was measured (Figure 2E). This analysis shows an alkaline shift of the activation pH dependence in G212E, small acidic shifts in G212D, G212Q, G212S, and G212T, and a strong acidic shift in G212F. The pH dependence of steady-state desensitization (SSD), the transition from the closed to the desensitized state that occurs without apparent opening at values slightly below pH 7.4, was then measured. To this end, oocytes were exposed during 55 s to a conditioning pH, before a short exposure to the stimulation pH5 to determine the fraction of channels that had not desensitized during this period. Figure 2F shows the normalized current amplitudes as a function of the conditioning pH. A pH of half-maximal desensitization (pHD_50_) value was determined from each experiment (Figure 2G). This analysis revealed a strong alkaline shift in the pH dependence of SSD in G212F, and acidic shifts in G212S and G212T.

Since the following computational analysis is based on crystal structures obtained with chicken ASIC1a, the WT and two mutations of this conserved Gly residue were also characterized in chicken ASIC1a. Mutation G213E induced an alkaline, while mutation G213D induced an acidic shift in the pH dependence of activation, analogous to what was observed in the context of human ASIC1a (Supplementary Figures S2A,B). These two mutations did not affect the pH dependence of SSD (Supplementary Figures S2C,D).

### Conformational Differences Between Gly212 Mutants Related to the Closed-Open Transition

Mutation of Gly212 to Asp and Phe, two residues with very different side chains, shift the pH dependence to more acidic values, thus hinder the channel opening, while the two rather similar residues Asp and Glu induce activation pH_50_ shifts in opposite directions. Thus, the analyses of the experimentally measured pH_50_ of activation sort the mutants in four groups (Figure 2E). To investigate conformational differences that affect ASIC activation, the MD simulations were carried out for one mutant for each of these groups, namely G212E (strongest alkaline shift), WT (sharing the same value as G212A), G212D (sharing similar values as G212Q, G212S, and G212T) and G212F (strongest acidic shift). The MD simulations were started from a homology model of human ASIC1a based on the crystal structure of the closed conformation of chicken ASIC1a (5WKU). Titratable residues were protonated to mimic pH5 conditions (section Materials and Methods). The simulation represents therefore the situation in which the channel is closed, and the pH is changed to an acidic value, to initiate ASIC activation. The relatively short MD simulations will not allow analysis of the closed-open transition, but should indicate whether the arrangement around residue 212 is different between ASIC1a WT and Gly212 mutants. For each construct, 3–4 independent trajectories of 200 ns duration were carried out, yielding a total simulation time of ∼ 3.4 μs (Supplementary Table S2).

#### Analysis of the Backbone Root-Mean-Square Deviation Highlights Differences Between Gly212 Mutants

**FIGURE 3:**
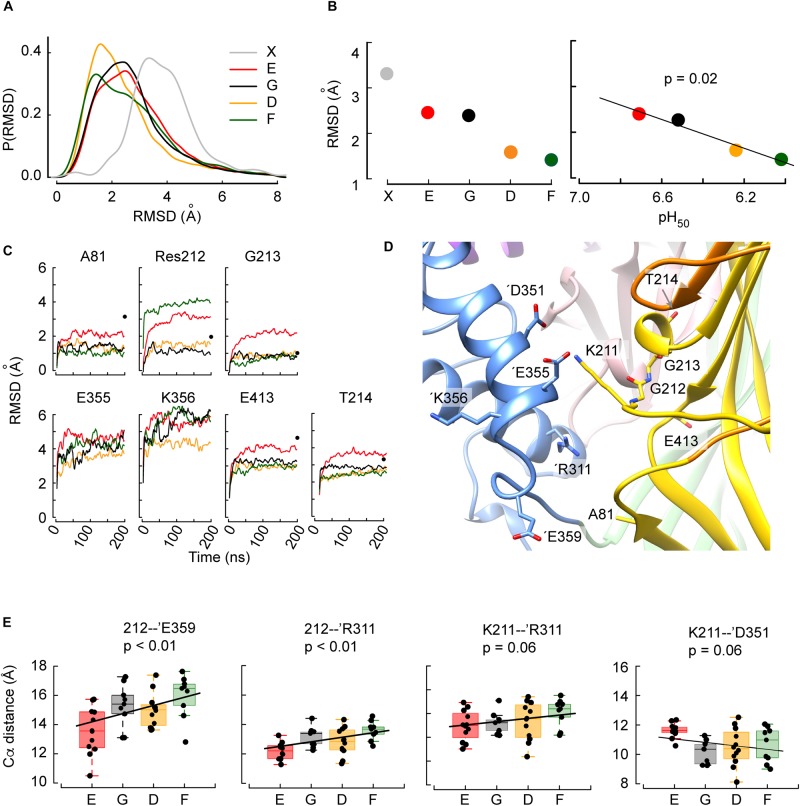
The structural deviations from the initial conformation and intersubunit distances obtained in MD simulations correlate with the pH_50_. **(A)** Distribution frequencies of the backbone root-mean-square deviations (RMSD, Å) of the residues located within 15 Å of residue 212, determined in MD simulations of G212E (red), WT (G212, black), G212D (orange), and G212F (dark green) of time points 50-200 ns relative to the initial structure, and between the closed (PDB code 5WKU) and open (4NTW) crystal structures (gray). **(B)** Left: Peaks of the distribution as a function of the mutants, sorted, from left to right, in the order of increasing acidic pH_50_. Right: Correlation between the pH_50_ and the RMSD for the investigated constructs. For comparison, the corresponding RMSD of the channel upon opening calculated from the crystal structures is shown in gray in **(A,B)**. **(C)** RMSD time series representations of the seven residues for which there was a statistically significant correlation between the RMSD and the pH_50_ ranking. The color code is as in **(B)**. Black dots at 200ns indicate the RMSD of the given residue between the closed and open structural model. For E355 and K356, the RMSD between the closed and the open structural model, not shown in the figure, is 8.9 and 11.4 Å, respectively. **(D)** Molecular representation of the area around position 212. The residues most affected by the mutation at position 212 and ’Arg311 are depicted in sticks and colored by atom types with nitrogen blue, oxygen red and carbon according to the domains they belong to. Note that the backbones of Gly212 and Gly213 are shown. **(E)** Correlations between mutants sorted in the order of increasing acidic pH_50_ and averaged distances between Cα atoms of Res 212 and ’Glu359, Res 212 and ’Arg311, Lys211 and ’Arg311, or Lys211 and ’Asp351. In all panels, the box plots display the median, the 25–75% range (box) and estimated minima and maxima. The linear regression parameters are calculated through least square estimation.

The analysis focused on the residues surrounding Gly212 within a distance of 15 Å, since conformational differences induced by the mutations are expected to be seen first in the direct vicinity of Gly212. This immediate surrounding involves 79 residues. To quantify the magnitude of the conformational changes during the transition from the closed to the open state, the backbone root-mean-square deviation (RMSD) from the initial structure (closed state) of these 79 residues was calculated as the average RMSD over the period between time points 50–200 ns. The distribution of the RMSD shows low values for G212D and -F, and higher values in WT and G212E (Figure 3A). For comparison, the RMSD of the homology model of the MitTx-opened ASIC1a (thus the completely opened channel) relative to the closed structure is indicated in gray. Its peak value was at ∼ 3.3 Å. The peak values of these distributions are plotted in Figure 3B. Interestingly, the observed trend E ∼ G > D ∼ F correlates with the pH_50_ ranking (Figure 3B), suggesting that G212E would undergo more conformational changes toward the opening of the channel than G212F. Since the alkaline shift of the pH_50_ observed in G212E indicates a facilitated opening of this mutant, the correlation between the RMSD and pH_50_ suggests that the MD trajectories have captured some of the initial conformational changes specific to the closed to open transition.

In order to better characterize the conformational changes related to the pH_50_ shifts, the residues within 15 Å distance of residue 212 were selected, whose RMSD in MD simulations was different between WT, G212E, G212D, and G212F. The aim of this selection was to identify the residues that cause the shifts in the above-described distributions of the RMSD. To this end, the RMSD of each of the 79 residues was plotted as a function of the pH_50_ ranking of Gly212 mutants (i.e., the sequence E-G-D-F), and a linear regression was calculated. Residues for which such a regression was significant were selected. This resulted in the identification of 7 residues (Ala81, 212, Gly213, Thr214, ’Glu355, ’Lys356, Glu413) located mostly in the palm of the same subunit and in the thumb of the adjacent subunit (Figure 3D). Plots of the evolution over the time of the MD simulation of the RMSD of these residues, in the four different constructs, are presented in Figure 3C. The plots indicate also for comparison the corresponding RMSD between the closed and open crystal structures (black dots at *t* = 200 ns). The comparison between these values and the values of the Glu212 trajectories in the different panels of Figure 3C suggests that, in terms of RMSD, the Glu212 trajectories are ∼ halfway of the open structure for most of these residues.

The study of the RMSD described above, which is essentially a proxy of the overall conformational changes, suggests that the MD trajectories have captured some of the initial conformational changes specific to the closed to open transition. In order to describe these conformational changes more in detail, atomic distances between an ensemble of 30 residue pairs surrounding residue 212 were systematically monitored in the four constructs investigated above. Ten of these distances involved Cα atoms. In order to perform comparisons that are (1) not biased by the size of the mutated residues (in the case of the position 212) and (2) that describe changes affecting the backbone atoms of the structure, the following analysis was carried out for these ten distances (Table 1). First, each distance was plotted as a function of the ranking of the constructs according to their pH_50_ (G212E, WT, G212D, G212F) and the correlation between the ranked constructs and the averaged distance over the trajectories was calculated. In four residue pairs, the slope between the Cα atom distances and the mutated residues was different from 0 (*p* < 0.01 in cases involving residue 212 and *p* ∼ 0.06 in cases involving Lys211). The distances of these residue pairs, 212 – ’Glu359, 212 – ’Arg311, Lys211 – ‘Arg311 and Lys211 – ’Glu351 (the suffix ’ indicates that the paired residue is located in a different subunit) are plotted in Figure 3E. Interestingly, these are intersubunit pairs, suggesting that the backbone structure and the interactions between subunits changed significantly as a function of residue 212. It is worth to note that the crystal structures indicate that the Gly212 – ’Glu359, Gly212 – ’Arg311 and Lys211 – ‘Arg311 distances decrease by ∼ 1.4 (from 15.8 to 14.3), ∼1.5 Å (from 11.9 to 10.5) and 2.0 Å (from 11.8 to 9.8), respectively, upon channel opening, while the 211 – ’Glu351 distance increases by ∼2.6 Å (from 10.2 to 12.8, 211 – ’Glu351). Thus, the G212E mutant was in all cases nearest to the open structure, suggesting that this mutation facilitates channel opening, leading thereby to an alkaline shift in the pH dependence of activation. These data suggest therefore that the G212E mutation, which shifts the pH dependence of activation to more alkaline values, may lead to an approach of the subunits at the level of position 212, similarly, to the crystal structures. On the other hand, the first three distances from left in Figure 3E are longest with the G212F construct, which is consistent with this mutation hindering channel opening. In all comparisons, the WT occupies an intermediate state, consistent with the idea that Gly at position 212 neither builds salt bridge interactions attracting the neighboring subunit, as G212E does, nor hinders the movement between subunits, as the bulky G212F does.

**TABLE 1:** Distances between Cα atoms of residues surrounding residue 212 in crystal structures and MD simulations starting from the closed ASIC1a model.

**Residue 1**		**Residue 2**		**Closed**	**G212F**	**G212D**	**WT**	**G212E**	**Open**
		
83	SER	212	–^a^	10.4	10.1	10.5	10.1	10.3	10.9
177	GLU	351	ASP	11.6	12.5	12.2	11.8	12.1	13.7
211	LYS	355	GLU	8.7	7.8	8.6	8.2	8.5	8.3
211	LYS	351	ASP	10.2	10.7	10.5	10.1	11.6	12.8
211	LYS	347	ASP	15.2	16.0	15.4	15.2	16.9	18.3
211	LYS	311	ARG	11.8	14.1	13.3	13.2	13.0	9.9
212	–	311	ARG	11.9	13.6	12.8	13.2	12.2	10.5
212	–	358	ARG	12.7	12.9	12.5	13.1	11.2	12.1
359	GLU	212	–	15.8	16.0	15.0	15.3	13.6	14.3
415	LEU	311	ARG	11.8	13.8	13.0	13.1	13.2	14.1

**The residues of the pairs are defined by their position and amino acid type. ^*a*^ “-” indicates position 212 with variable residue name depending on the investigated mutation. The numbers under the headings “G212F,” “G212D,” “WT,” and “G212E” are distances between Cα atoms of the indicated residues, in Å, calculated over the whole MD trajectories after discarding the first 20 ns, and averaged over 3–4 replications per mutant. The distances between the same* α *carbons were also measured in the closed and open structural models, as indicated.**

In summary, in the four (out of ten) distance measurements that could be ranked significantly in the order of the pH_50_ of activation, the “slope” correlated with the channel opening as inferred from the crystal structures. While some of the other compared pairs would be ranked differently (Table 1), it is noteworthy that none of the six corresponding regressions was significant.

#### Conformational Differences Between G212E and G212D

**FIGURE 4:**
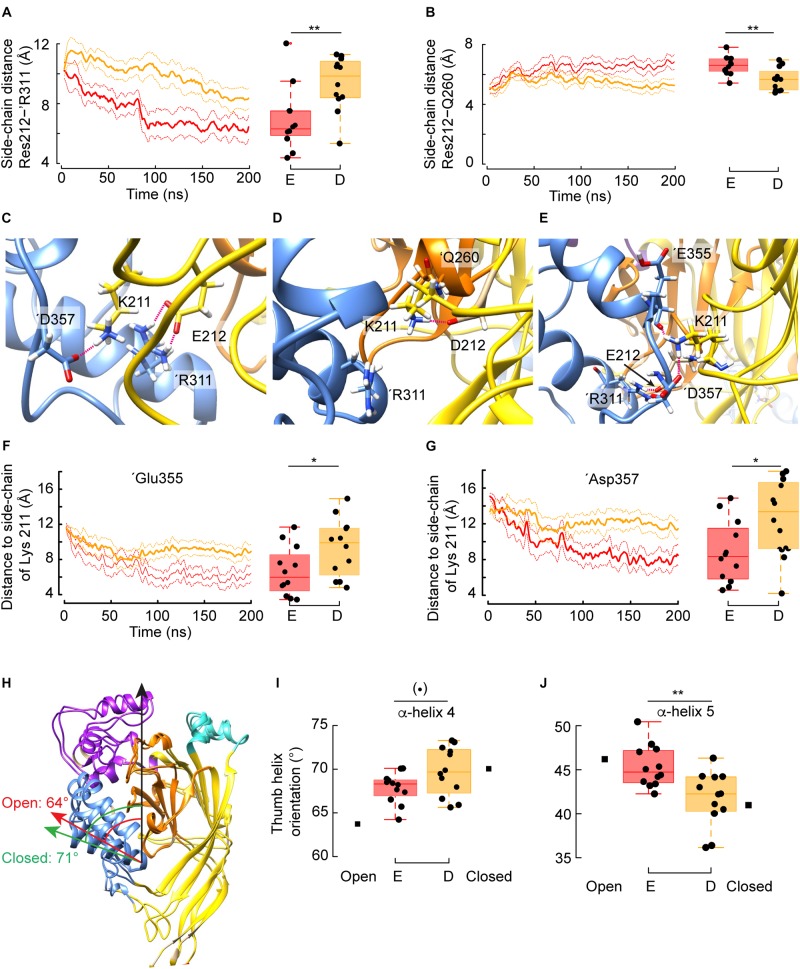
Mechanism by which Glu but not Asp at position 212 favors channel opening. **(A)** Averaged time series of the distance between the guanidium group of ’Arg311 and the carboxylic group of Glu212 (red) or Asp212 (orange). Right: Corresponding quantification over the whole trajectory (first 20 ns discarded). Glu212: *n* = 12, 4 independent simulations, Asp212: *n* = 9, 3 independent simulations. **(B)** Averaged time series of the distance between the side chain of Gln260 and the carboxylic group of Glu212 or Asp212. Right: Corresponding quantification over the whole trajectory (first 20 ns discarded). **(C)** Typical molecular representation in the context of a simulation with G212E, in which Glu212 and ’Arg311 form two salt bridges, while Lys211 interacts with ’Asp357. **(D)** Typical molecular (representation in the context of a simulation with G212D. Because Asp212 is oriented toward the β-ball and interacts with ’Thr260, Lys211 cannot reach to ’Arg311, and forms a salt bridge with Asp212. **(E)** Typical molecular representation (G212E) showing the network of electrostatic interactions involving Lys211 with ‘Glu355 and ‘Asp357 co-occurring with a salt bridge between G212E and ‘Arg311. **(F)** Averaged time series of the distance between the side chain of Lys211 and the carbonyl oxygen of ‘Glu355 in the context of G212E or G212D. Right: Corresponding quantification over the whole trajectory (first 20 ns discarded). **(G)** Averaged time series of the distance between the side chain of Lys211 and the carboxylic group of ‘Asp357 in the context of G212E or G212D. Right: Corresponding quantification over the whole trajectory (first 20 ns discarded). **(H)** Overlay of one subunit of the closed (PDB code 5WKU) and open (4NTW) crystal structure. The green arrow indicates the orientation of α-helix 4 in the closed, while the red arrow shows the α-helix 4 orientation in the open structure. The values (degrees) of the angles formed by the α-helix 4 and the principal axis of the protein, marked by a black arrow, are indicated. **(I,J)** Averaged orientations (as in panel **H**) of α-helix 4 **(I)** and α-helix 5 **(J)** in the case of trajectories with the G212E or G212D mutants. For comparison, the corresponding angle values extracted from the closed or open crystal structures are indicated. Figure color code: Glu212 red, Asp212: orange. In all panels, the box plots display the median, the 25–75% range (box) and estimated minima and maxima. The results of statistical analyses are presented as follows: (**⋅**): marginally significant, *p* < 0.1; *p < 0.05; **p < 0.01.)

Given that Asp and Glu are both acidic residues, it was surprising to find that the G212E and -D mutations have opposed effects on the pH dependence of activation. The analysis of the MD simulations shows that the side chain distance between residue 212 and ’Arg311 of the adjacent subunit – a readout for the intersubunit distance at this level – is shorter in G212E as compared to G212D (Figure 4A, left: time course, right: summary of the averaged trajectories, *n* = 9–12, *p* = 0.02). The mean distance of ∼ 6 Å indicates that Glu at position 212 tends to form a salt bridge with ’Arg311. The side chain of Glu is ∼ 1.5 Å longer than the side chain of Asp, which seems sufficient for the negatively charged carboxylic group to sense the positive charge of the ’Arg311. This Glu212-’Arg311 salt bridge constitutes an additional interaction between the two subunits, by which Glu212 may pull the base of the thumb of the adjacent subunit toward the palm. The distance between the Gly212 and ’Arg311 Cα atoms was significantly (*p* = 0.02) shorter by 0.7Å in the presence of Glu212 (12.2 vs. 12.9Å, Figure 3E). This is reminiscent of the crystal structures, where the same distance decreases by ∼ 1.5Å upon channel opening (from ∼12 Å in the closed to ∼10.5Å in the open state). Thus, with regard to the distance between the thumb α-helix 4 and the palm domain, the simulations performed with the G212E resemble more the open structure than those performed with the G212D. For comparison, the same intersubunit distance was not affected in the WT, and was even higher than in the closed structure in the case of G212F (Figure 3E). Molecular representations of this zone illustrate the salt bridge formation between residue 212 and ’Arg311 in G212E but not G212D (compare Figures 4C,D).

The distance between Gly212 and ’Gln260 of a neighboring subunit is increased in the open, as compared to the closed structure. In MD simulations, the side chain distance between the residue at position 212 and ’Gln260 was consistently shorter in G212D, and of a distance that indicates the formation of a salt bridge between these residues, which did not, or only rarely occur with G212E (Figure 4B). This interaction, which likely stabilizes the closed state conformation in G212D, is illustrated in Figure 4D.

Lys211 has been shown to be important for channel activation (Lynagh et al., 2018). The deletion of Lys211 induced a pronounced acidic shift of the pH_50_. To evaluate whether these two mutations affected differently the position of Lys211, which interacts with the adjacent subunit, distances between Lys211 and two residues of the ’α5 α-helix of the acidic pocket in the neighboring subunit were measured. According to the crystal structures, the distance between the Lys211 side chain and the ’Asp357 side chain and the ’Glu355 backbone oxygen residue decreases upon opening. Interestingly, for both distance measurements, G212E displays distances similar to the open conformation, whereas G212D has a behavior that is reminiscent to the closed conformation (Figures 4F,G). Thus, the data suggest that the mutant G212E promotes activation by the formation of salt bridges of Lys211 with ’Glu355 and ’Asp357, respectively. This network of electrostatic interactions between the lower thumb and both residues G212E and K211 is illustrated in Figure 4E. In view of these observations, it is likely that the Glu212-’Arg311 salt bridge formation described above facilitates the formation of a second interaction connecting Lys211 with any of these two acidic residues of the lower thumb. On the other hand, the crystal structures suggest that the distances between the Cα atoms of Lys211 and ’Asp347 and ’Asp351, respectively, increase by 3.1 and 2.6 Å upon channel opening. The corresponding distances were also significantly higher by 1.6 and 1.2 Å, respectively (*p* = 0.006 and *p* = 0.003) in the simulations conducted with G212E as compared to the simulation carried out with G212D, further suggesting that some of the structural changes specific of the channel opening have been captured by the MD and that transitions toward opening occur earlier in G212E than in G212D. These distances, however, are large (∼15Å) and thus do not implicate direct interactions. However, they further confirm the difference between G212E and G212D in the conformational changes of the closed-open transition.

Since the simulations had captured features characteristic of the closed to open transition in terms of overall structural changes (RMSD), side-chain interactions (salt bridges) and also Cα distances, and since these features correlated with the behavior of the mutants in terms of shift in the pH_50_ of activation, we wondered whether local sub-domain movements would have been captured as well. The crystal structures suggest that α-helices 4 and 5 of the thumb domain undergo a reorientation upon channel opening. Thus, the angle formed between α-helix 4 and the protein principal axis decreases from ∼ 71 to 64° upon opening (Figure 4H), whereas the relevant angle of α-helix 5 increases from ∼ 41 to 46°. When position 212 was a Glu residue, both thumb α-helices reoriented toward the open state, and in this the trajectories differed from trajectories conducted with G212D (*p* = 0.06 and 0.004, respectively, for α-helix 4 and α-helix 5). This observation indicates that the above described additional interaction between Glu212 and ‘Arg311 suffices to reorganize local substructures within a few hundreds of nanoseconds.

#### Conformational Differences Between G212F and G212E

**FIGURE 5:**
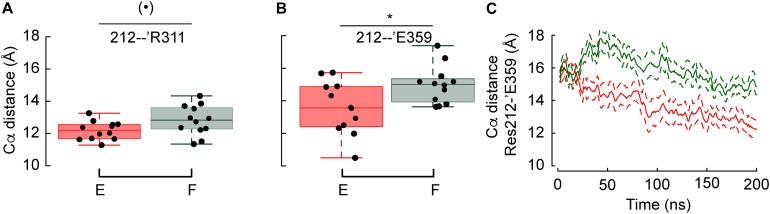
Phe at position 212 prevents the lower thumb to approach the palm domain. **(A,B)** Average distances between the Cα atoms of Res. 212 – ’Arg311 and Res. 212 – ’Glu359 in G212F and G212E. **(C)** Averaged time series of the distances between the Cα atoms of G212E and G212F, respectively, and ‘Glu359. Figure color code: Glu212 red, Phe212: green. The box plots display the median, the 25–75% range (box) and estimated minima and maxima. The results of statistical analyses are presented as follows: (**⋅**): marginally significant, *p* < 0.1; *p < 0.05.

Since these two constructs show the most diverging pH dependence of activation among the tested mutants, the position of residues in the proximity of residue 212 was compared. Two out of ten measured distances involving Cα atoms (see section Analysis of The Backbone Root-Mean-Square Deviation Highlights Differences Between Gly212 Mutants) were significantly different between G212F and G212E. As shown in Figures 5A,B, the distances between the Cα atoms of residue pairs 212-’Arg311 and 212-’Asp359, respectively, are longer by 1.4 and 2.4Å when position 212 is occupied by a Phe instead of Glu. The analysis of the crystal structures implies that these two distances involving movements of the lower thumb domain decrease by 1.5 and 1.4 Å, respectively, upon channel opening. This indicates that the acidic shift observed when position 212 is occupied by a Phe is due to the fact that Phe prevents the lower thumb to approach the palm domain. The Phe and Glu side-chain are of similar length, with Phe being ∼0.4 Å longer. Thus, the difference between the Cα distances observed here, of magnitude 1.4 (Res212-’Arg311) and 2.4 Å (Res212-’Glu359) reflect at least partly an attraction, respectively, a repulsion of the lower thumb domain in the case of G212E and G212F, respectively. Interestingly, the evolution through time of the 212-’Asp359 average distance (15 and 9 replicates for G212E and G212F, respectively) suggests that the lower thumb moves toward the palm in both cases, but with a delay in the case of G212F (Figure 5C).

### Strong Dependence of Current Decay Kinetics on the Residue at Position 212

**FIGURE 6:**
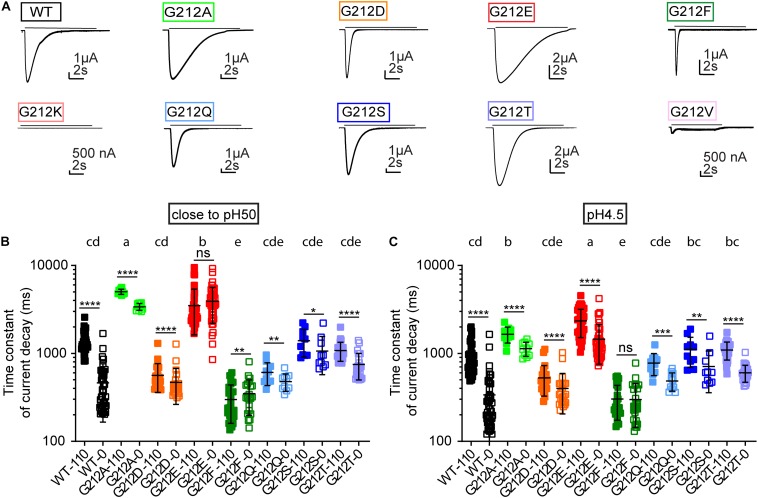
Kinetics of current decay. **(A)** Representative current traces of the WT and the Gly212 mutants. Conditioning pH7.4 (pH7.8 for G212F) was applied for 50 s followed by a 10 s step stimulation at pH6 (indicated by the horizontal line). **(B)** Time constant of current decay at 0 and 110 mM Cl^–^. In the 0 mM Cl^–^ condition, Cl^–^ was replaced by SCN^–^. ASICs were stimulated by a pH that was close to their pH_50_. Mutants that do not share the same letters are significantly different in the condition of 110 mM Cl^–^ (*n* = 10–43, *p* < 0.05). **(C)** Time constant of current decay at pH4.5, at 0 and 110 mM Cl^–^. Mutants that do not share the same letters are significantly different in the condition of 110 mM Cl^–^ (*n* = 10–43, *p* < 0.05). Measurements at 0 and 110 mM Cl^–^ were performed on the same oocyte. Ordinary One-Way ANOVA followed by Tukey’s HSD test for multiple comparisons was used for statistics, paired *t*-tests were used to compare 0 and 110 mM Cl^–^ for each mutant; *p < 0.05; **p < 0.01; ***p < 0.001; ****p < 0.0001; ns, non-significant.

We had previously shown that the current decay kinetics measured at close to physiological Cl^–^ concentrations were accelerated in the G212D mutant (Vaithia et al., 2019). The representative current traces in Figure 6A emphasize the important role of residue 212 for the current decay kinetics. These kinetics were quantified by fitting to a single exponential, at a pH that was for each mutant close to its pH_50_ value (Figure 6B) and at a very acidic pH (pH4.5; Figure 6C). The currents were measured from the same oocytes in solutions containing 110 mM Cl^–^, and in solutions in which the Cl^–^ was entirely replaced by SCN^–^. At pH4.5 and 110 mM Cl^–^, G212E showed the slowest current decay kinetics. The current decay of G212A was faster than G212E, and slower than the WT. Most other mutants had similar current decay kinetics as the WT, except for G212F, whose τ values were smaller than the WT values. The pattern of the τ values as a function of the mutant and Cl^–^ condition was similar in the two pH conditions, except for G212A, for which the τ was more slowed relative to the other mutants at the pH close to the pH_50_ (Figures 6B,C). Although the G212D mutant did, in the context of this comparison with many other mutants, not display significantly faster current decay kinetics with 110 mM Cl^–^ than the WT, there was a clear tendency for faster current decay, with p values of 0.058 and 0.053 at pH close to pH_50_ and at pH4.5, respectively.

Gly212 is located in close proximity of a predicted Cl^–^ binding site in open and desensitized ASIC1a (Jasti et al., 2007; Baconguis and Gouaux, 2012; Yoder and Gouaux, 2018). Interestingly, the differences in current decay kinetics between WT and G212D were much smaller when the extracellular Cl^–^ was replaced by SCN^–^ (Vaithia et al., 2019). In the present study, the current decay kinetics were for most mutants – except for G212E at pH close to pH_50_, and G212F at both pH conditions – faster with the solutions containing SCN^–^ (Figures 6B,C). Thus, the substitutions at position 212 preserved the regulation of the decay kinetics by the anion type. The ratio of the time constants (τ_110 mM Cl_- /τ_110 mM SCN_-) was, however, 3.5 ± 1.3 (*n* = 40) with the WT at pH4.5 and very similar close to the pH_50_, while it was < 2 for all other mutants (*n* = 10–44), thus this ratio was significantly higher in the WT compared to all other substitutions at both pH conditions (*p* < 0.0001; compare the values in Figures 6B,C for the different anion conditions).

Two mutations of Gly213, the homologous residue of chicken ASIC1a, were studied. At 110 mM Cl^–^, the G213E mutation induced a substantial slowing of the current decay kinetics at a pH close to its pH_50_, but not at pH4.5 (Supplementary Figures S2E,F), and the kinetics of G213D were very similar to those of the WT. In the three cASIC1a constructs, the current decay kinetics depended in all, except one condition (G213E, pH close to pH_50_) on the type of anion present in the solution. In cASIC1a, the τ_110 mM Cl_-/τ_110 mM SCN_- ratio of the WT was 1.5 ± 0.7 (*n* = 23) at pH4.5, and similar at a pH close to the pH_50_, while this ratio showed a tendency to higher values in the mutants (*n* = 22–24), and was significantly increased at pH4.5 by the G213D mutant. Taken together, the experiments with cASIC1a show a qualitative conservation of the effects of mutations of this residue between human and chicken ASIC1a. The effects on the current decay kinetics are, however, smaller in cASIC1a.

The functional data show a difference in the anion effect between the WT and all mutants. The acidic substitutions did, however, not behave differently from Phe and Thr with regard to the modulation of the current kinetics by anions. Since there are basic, but not acidic residues located close to position 212, the pKa of Glu or Asp side chains at position 212 is likely low, resulting in negatively charged side chains.

### Conformational Differences Between Gly212 Mutants Related to the Open-Desensitized Transition

To identify the structural basis of differences in current decay kinetics between Gly212 mutants, MD simulations were carried out for the WT (Gly212) and for four mutants, including G212E, G212D and G212F studied above for the closed-open transition, and in addition G212T, which represents a mutant with properties that are in-between the extremes. The current decay corresponds to the transition from the open to the desensitized state. Intriguingly, there exist only very subtle differences between the open and desensitized structures in this part of ASIC (Baconguis et al., 2014). MD simulations of ∼ 200 ns duration each (Supplementary Table S2) were carried out with homology models of either open or desensitized hASIC1a carrying Gly, Asp, Glu Phe or Thr at position 212, embedded in a lipid bilayer (see section Materials and Methods). To identify structural differences between mutants, local and global rearrangements in the vicinity of residue 212 were explored. Local changes due to the mutations were determined by the analysis of the orientation of side chains, while global conformational changes were investigated through cross-correlation study of the Cα atom coordinate trajectories.

#### Different Side Chain Rearrangements Induce a Repositioning of the Thumb α-Helix 4

**FIGURE 7:**
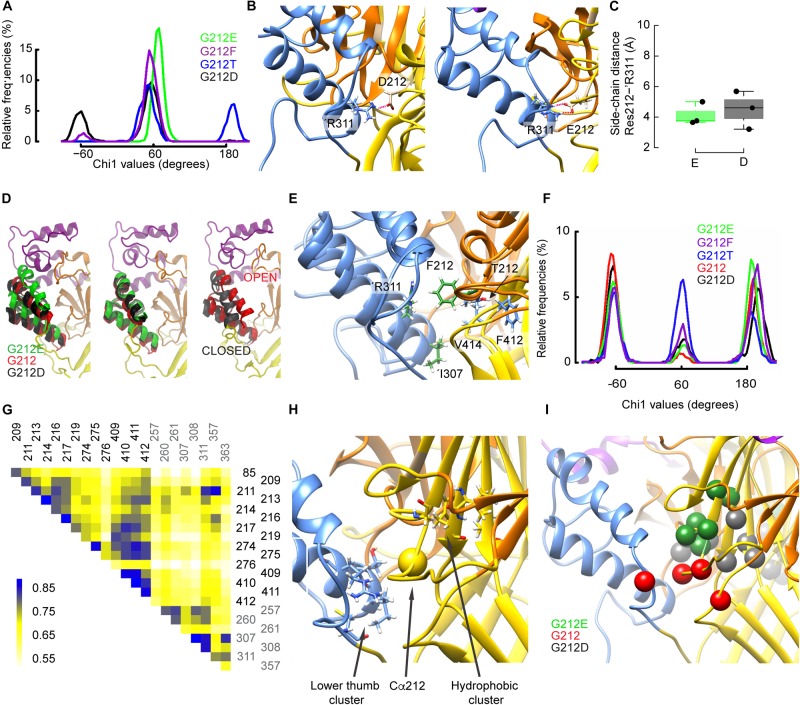
Gly212 mutants induce conformational changes that affect desensitization. **(A)** Probability density of χ1 orientations of residue 212 in the indicated mutants, from the last 150 ns of MD trajectories started from the desensitized structure. **(B)** Molecular representations of ASIC1a containing G212D (left) and G212E (right) showing the heavy atoms of selected residues. The ASIC domains are colored as follows: palm: yellow, thumb: blue, beta ball: orange, knuckle: cyan. Red dashed lines indicate salt bridges **(C)** Average distances between the side-chain atoms of G212E and G212D, respectively, and ’Arg311. **(D)** Comparison of the thumb helix reorientation of WT (Gly212), G212D, and G212E where the mutated residue at position 212 has a χ1 value of ∼-60 (left panel) or +60 (middle panel) with a superposition of the open and closed crystal structures (right panel). For the WT, a typical conformation was taken. **(E)**, Superposed molecular representations of G212F and G212T. Coloring as in **(B)**. Note how ’Arg311 is repelled by Phe212. **(F)** Probability density of χ1 orientations of residue ’Asp357 in the indicated mutants, from the last 150 ns of MD trajectories, started from the desensitized structure. **(G)** Example of a correlated motion matrix in the vicinity of the residue 212. The matrix elements of the cross-correlation heat map are colored according to the scale bar shown on the left side of the plot. Shown here is the analysis of MD simulations based on the desensitized model of WT hASIC1a. **(H,I)** Molecular representation in the vicinity of the residue 212 highlighting clusters of simultaneously moving residues, as identified through cross-correlation matrix analysis. **(H)** Residue pairs with high cross-correlation values in all constructs. **(I)** residue pairs whose correlation was high with specific substitutions at position 212.

The χ1 angle describes the orientation of a side chain relative to the peptide backbone. The χ1 angle of different residues close to the residue 212 was monitored to describe side chain re-orientations induced by the mutations. At the start of the simulations, all mutated residues at position 212 were oriented at a χ1 of ∼ 180°. Figure 7A shows the χ1 values of the mutants obtained during the last 150ns of the trajectories started from the desensitized state. While the Glu side chain systematically adopted an angle of ∼60°, the Asp side chain rotated during some periods (∼32% of simulation time) to a value ∼-60°, thus to a downward orientation (Figure 7A). Contrary to the closed-to-open transition, both acidic side chains formed a salt bridge with ’Arg311, which is located in the thumb of the adjacent subunit, and this interaction persisted during the whole simulations (Figure 7B). Figure 7C displays such salt bridges observed between ’Arg311 and Asp212 or Glu212. Interestingly, the downward movement of the Asp212 side chain to an orientation of ∼ -60° coincided with a large rearrangement of the adjacent thumb α-helices (Figure 7D, left panel; the middle panel shows the orientation for χ1 of ∼ +60°) that resembled their position in the ASIC closed state (Figure 7D, right panel). During the periods in which Asp212 was oriented at a χ1 of ∼+60°, the position of the thumb α-helices was very similar to that of the G212E mutant (Figure 7D, middle panel, and Supplementary Figure S3G). In the WT (G212) simulations, thus in the absence of any acidic side chain at position 212, ’Arg311 formed hydrogen bonds with ’Glu315 or interacted with Lys211 via water-mediated hydrogen bonds, and the thumb α-helices lied in between the above mentioned two positions (Figure 7D). The thumb position of G212E, WT (G212), and G212D in MD simulations correlated with the experimentally observed current decay kinetics: the higher the thumb α-helices in the MD simulations, the slower the experimentally determined current decay kinetics. The downward orientation change of Asp212 also placed its carboxyl group close to residues of the β1–β2 loop in the lower palm, resulting in some parts of the simulation in a proximity of <3 Å between the hydroxyl group of Ser83 and the carboxyl of Asp212. The analysis of the χ1 angle also shows that the side chain of G212F adopted a position of ∼60°, and that the Thr side chain remained during parts of the simulation, mostly in simulations starting from the desensitized state, at the initial value of ∼180° (Figure 7A and Supplementary Figure S3A). Visual inspection of the trajectories revealed that Thr212 interacted essentially with residues of the same subunit, specifically with Glu413 and Val414 (during periods where χ1 ∼60°), or with backbone atoms of Phe412 (χ1 ∼ 180°), whereas Phe212 was often found squeezed in between ’Arg311 and either Val414 or Ile307 (Figure 7E). Contrary to Asp212, these interactions of Phe212 with the adjacent thumb domain did not induce any large conformational changes during the simulation time.

A recent study suggests that Lys211 may contribute to ASIC activation through interactions with residues at the base of the thumb domain (Lynagh et al., 2018). The χ1 value of Lys211 switched in all substitutions between the three possible rotamers defined by values of ∼+60°, ∼-60° and 180° (Supplementary Figures S3C,D). Lys211 undergoes hydrogen bonding with backbone atoms of ’Asp357 and ’Cys361 in the loop connecting the thumb domain to the palm. It was therefore tested whether these residues may be affected by the mutations at position 212. Indeed, the occurrence of the side chains of both residues at ∼-60° was three to nine times higher with Glu than Phe at position 212, and was also high in the WT (Gly212; Table 2, Figure 7F and Supplementary Figures S3B,E–F). The previous section dedicated to the closed-open transition showed that in the presence of Glu at position 212, interactions between Lys211 and ’Asp357 are implicated in the channel opening. In the ∼-60° orientation, ’Asp357, which is located near the lower part of α-helix 5, is oriented toward the palm domain. This position is hindered by the bulky and hydrophobic Phe at position 212, so that any interaction of ’Asp357 with Lys211 becomes unlikely. In the presence of Phe at position 212, the mobility of ’Asp357 is considerably impaired, as exemplified by the comparison of time series involving Phe and Thr at position 212 (Supplementary Figures S3H,I). The differential effect of G212E and G212F on ’Cys361, however, remains difficult to interpret, since ’Cys361 is located relatively far from the residue 212 and forms a disulfide bond with ’Cys314 located in α-helix 4.

**TABLE 2:** Proportion (%) of residue ’Asp357 and ’Cys361 with χ1 values of ∼-60° in MD simulations with different residues at position 212.

**Res212**	**’Asp357**	**’Cys361**
E	46.1	38.2
T	30.7	20.9
G	40.1	27.2
D	37.4	24.2
F	16.8	4.3
E/F*	2.8	8.9

For this analysis the total simulation time, except for the first 50 ns, was used. Values are from simulations started from either the open or the desensitized state model, obtained at 1 frame/ns in each subunit. The number of frames in which χ1 was∼60° was divided by the total number of frames. *Ratio of the number of frames in the E vs. the F mutant.

Taken together, these observations, obtained in simulations starting from the open conformation, suggest that Glu at position 212 enforces intersubunit interactions and that substitutions at this position may differently affect the position of the thumb α-helices. This may involve a direct interaction, through a salt bridge with ’Arg311, and/or changed interactions between Lys211 and the backbones of ’Asp357 and ’Cys361.

#### Cross-Correlation Analysis of Movements in the Proximity of 212

To investigate global conformational changes, the correlation of movements (Grant et al., 2006; Sethi et al., 2009; Gaieb and Morikis, 2017) of 40 residues located within ∼8–10 Å of the Cα atom of residue 212 were analyzed during MD simulations that started from the open and desensitized conformation. Using approaches described previously (Cunningham, 2012; Gaieb and Morikis, 2017), displacements of the Cα atoms of these residues were monitored and the correlation between movements of different residues was analyzed on the G212E, G212D, and WT channels. It is expected that the cross-correlation over time will be high if the pairs interact tightly. The analysis was only performed between residues that were at least three residues distant from each other in the primary structure. A typical cross-correlation matrix of the 22 best-scoring residues is shown in Figure 7G for G212. A two step-procedure was used to select residue pairs that displayed a strong covariance, which was reproducible over several independent trajectories. This analysis identified first two clusters of residues as constituting an intra-subunit network common to the three tested constructs (Figure 7H and Supplementary Table S3). The first cluster consisted of ’Ile307, ’Thr308, ’Arg311, and ’Cys363 at the lower end of the thumb (pairs ’307–’363, ’307–’311, ’308–’311), and a partially hydrophobic cluster, comprising Gly217, Glu219, Ala274, Asp409, Ile410, and Phe411 in the center of the upper palm (pairs 217–410, 219–409, and 274–411; Figure 7H). The residues within each of these clusters undergo coordinated movements in all tested constructs. Note that these clusters do not comprise intersubunit pairs. Next, pairs of correlated residues unique to each of the three tested constructs were identified (Figure 7I). With G212E, a cluster of residues within the β5–β6 palm loop and between this loop and Asp409 of the palm (Gly213, Thr214, Asn216, Gly217, Glu219, Asp409) displayed a high correlation. These pairs were located above residue 212, and in the same subunit. The analysis of the WT (Gly212) trajectories highlighted an intra-subunit pair, Leu85 of the β1–β2 linker of the palm and Thr209 of the β5–β6 palm loop, and the intersubunit pair of Lys211 and ’Asp357 in the thumb α-helix 5, illustrated by the high cross-correlation value in the heat map (Figure 7G). The analysis of the G212D trajectories identified pairs within the β7–β8 β-ball loop (Phe257, Glu260, Leu261) and between the β strands 9 and 11 of the palm (Ala274, Cys275, Ile410, Phe412), thus in both cases within a subunit. The section “Conformational Differences Between Gly212 Mutants Related to the Closed-Open Transition” has shown the importance of intersubunit interactions in the context of channel opening. The absence of strong intersubunit crosscorrelation in both G212D and G212E in the analysis of the open-desensitized transition suggests that other factors, which were not identified in our analysis, might affect the rate of desensitization. This may be due in part to the very small differences in conformation of open and desensitized structures in this area. Nevertheless, this analysis uncovers several patterns of correlated movements in Gly212 mutants.

## Discussion

We showed in this study that substitutions of the conserved Gly212 change the pH dependence, and the kinetics of desensitization of ASIC1a currents, and do not affect ASIC expression at the plasma membrane. Gly212 is located in the palm, at a subunit interface in the proximity of the lower ends of the thumb α-helices 4 and 5. Comparison of the crystal structures of cASIC1a in the closed, open and desensitized states indicates a substantial rearrangement of this interface in the transition from the closed to the open, and almost no changes in the transition from the open to the desensitized state (Gonzales et al., 2009; Baconguis et al., 2014; Yoder et al., 2018). Several residues close to Gly212 are involved in intersubunit interactions, among them Lys211 and ’Arg311. MD simulations starting from the closed conformation revealed that mutations of Gly212 affect intersubunit interactions, most importantly salt bridges that either favor or hinder the closed-open transition, as well as the positioning of the thumb α-helices. MD simulations starting from the open conformation suggest that Gly212 mutations affect intersubunit interactions and the position of the thumb α-helices, leading in the case of Gly212 and G212E to slower desensitization than with G212D and G212F.

### Functional Importance of Intersubunit Interactions Close to Gly212

Interactions between the β-ball of one, and the thumb of the neighboring subunit were previously suggested by Cys mutations and subsequent chemical modification (Krauson and Carattino, 2016). It was shown that the mutations and modifications affected mostly the current decay kinetics; from Cys accessibility experiments it was concluded that in the resting state the β-ball and neighboring thumb reside apart, but that they move closer to each other upon acidification (Krauson and Carattino, 2016). Support for a role of the immediate environment of Gly212 in ASIC1a activation comes from the observation that an engineered disulfide bond in this region (R175C-’E355C) decreased the current amplitude and induced an acidic shift in activation pH dependence (Gwiazda et al., 2015), and that deletion of Lys211 also induced a strong acidic shift of the activation pH dependence (Lynagh et al., 2018). Disulfide bond formation in the close vicinity, between chicken ASIC1a T84C and N357C also strongly reduced current amplitudes (Yoder and Gouaux, 2018).

### Differences in RMSD Relative to the Closed Conformation in Gly212 Mutants Are Related to Activation pH_50_

MD simulations starting from a closed conformation were used to study possible structural differences between Gly212 mutants that may be important for ASIC activation. Analysis of the RMSD relative to the closed conformation in the proximity of residue 212 showed that the RMSD was smaller in MD simulations as compared to the MitTx-opened structural model, and that there was a positive correlation between the RMSD and the pH_50_, suggesting that mutants with more alkaline pH_50_ can adopt within a few hundreds of nanoseconds a conformation that is close to the open conformation, while mutants with acidic pH_50_ keep during this time conformations close to the resting conformation.

### Mutant-Specific Intersubunit Salt Bridges Favoring Closed or Open Conformations

The analysis of distances between Cα atoms of residues in simulations starting from the closed conformation highlighted an approaching between subunits at the level of position 212. Such an approaching is indeed predicted to occur upon opening, from the comparison of the distance between the Cα atoms of Lys211 and ’Arg311 in the closed (11.8 Å) and open crystal structures (9.9 Å) (Yoder et al., 2018). This intersubunit approaching was facilitated by the G212E mutation (high pH_50_) and hindered by the G212F mutation (low pH_50_). To understand the basis of the functional differences between the Glu and Asp substitutions, a number of side chain distances were followed over time, and it was found that the Glu sidechain at position 212 allowed the formation of a salt bridge with ’Arg311, which favors opening, while Asp at position 212 formed interactions with ’Gln260, favoring the closed conformation. In addition, Lys211 was able to approach the carbonyl of ’Glu355 and the carboxylic functional group of ’Asp357 residues of the neighboring subunit. These two additional interactions between G212E in the palm and the lower part of the thumb result in a reorientation of the thumb reminiscent of the X-ray open structure (Figures 4H–J). Taken together, this analysis shows that the small size difference of Asp and Glu sidechains suffices at this particular position to induce marked changes of intersubunit interactions, favoring channel opening in the case of G212E. While the negatively charged G212E attracts the basis of the thumb, the bulky and hydrophobic G212F hinders any movement of the basis of the thumb toward the palm, resulting in a slowing of the channel opening. Gly at position 212 does not induce any steric hindrance of movements in this area, nor does it exert electrostatic side chain interactions. Since the presence of Gly in the β5–β6 palm loop is expected to confer flexibility to this loop, it may help to accommodate any structural changes. Together, this may lead to the observed pH_50_ value of the WT that is intermediate between that of G212E and G212F.

### Relative Position of Thumb α-Helices, and Intra- and Inter-Subunit Interactions Likely Influence the Current Decay Kinetics

The analysis of MD simulations based on open and desensitized structural ASIC1a models highlighted differences between channels with substitutions at position 212, which may underlie the observed diversity in current decay kinetics. We observed that the higher the thumb α-helices were positioned, the slower were the kinetics of current decay for ASIC1a WT and the G212E and G212D mutants. In the case of G212D, the changed position of the thumb α-helices correlated with a difference in the χ1 angle of residue 212. Second, the analysis of correlated movements points also to differences in intra- and intersubunit interactions between the mutants and suggests that Gly212 does not hinder intersubunit interactions. The correlation analysis also suggests that the β5–β6 palm loop that contains residue 212 moves as a rigid body in G212E, and that this movement is correlated to that of residues further up in the palm. Together, this may be at the origin of the slowed current decay in G212E. The large side chain of a Phe residue at position 212 adopted a similar χ1 angle as Asp212, was frequently found between ’Arg311 and hydrophobic residues, and generally decreased the intersubunit interactions, which may be at the origin of the faster current decay kinetics.

### The Dependence of the Current Decay Kinetics on the Anion Type in Functional Experiments Is Maintained in All Gly212 Mutants

A Cl^–^ binding site in close proximity to Gly212 has been described in crystal structures of open and desensitized cASIC1a. Since the chloride concentration is known to affect the ASIC current decay kinetics (Kusama et al., 2010, 2013), which is also influenced by the type of residue at position 212, the residue 212 may interact with the bound Cl^–^ ion. The earlier studies had shown that mutations of residues forming the predicted Cl^–^ binding site (corresponding to Lys211, ’Arg311, and ’Glu315 in hASIC1a) disrupt the modulation of the current decay kinetics if introduced in ASIC1a (Kusama et al., 2010), and have a partial, or no effect if introduced into ASIC2a or ASIC3, respectively (Kusama et al., 2013). In the first of these two studies it was concluded that both, SCN^–^ and Cl^–^ bind to the same binding site in ASIC1a, and that SCN^–^ even binds with higher affinity than Cl^–^, but cannot induce a modulatory effect and therefore acts as a kind of competitive antagonist (Kusama et al., 2010). This study left the question open of how Cl^–^ binding slowed the current decay kinetics. A Cl- binding site at this position was found in the open and desensitized but not in the closed ASIC1a structure (Jasti et al., 2007; Gonzales et al., 2009; Baconguis and Gouaux, 2012; Baconguis et al., 2014; Yoder and Gouaux, 2018; Yoder et al., 2018). Our functional analysis shows that the modulation of the current kinetics by Cl^–^ was maintained in most substitutions of residue 212. The functional analysis showed, however, also that the ratio of the time constants (τ_110 mM Cl_- /τ_110 mM SCN_-), thus the amplitude of the effect of anion binding, was highest in WT (Gly212). Together, this suggests that substitutions at position 212 affect not the binding of Cl^–^ itself, but, due to the changed intra- and intersubunit interactions in this area, the consequences of Cl^–^ binding.

## Conclusion

Gly212 is located at an intersubunit contact site. We show here that mutations of Gly212 affect the ASIC1a pH dependence and current decay kinetics. These effects are caused by differences in inter- and intrasubunit interactions, among others the formation of salt bridges, in this area, which undergoes subtle conformational changes during desensitization but substantial changes during opening. Our analysis improves the understanding of the mechanisms controlling ASIC activity. This study opens a way for the development of a new class of ASIC modulators that would target the identified interaction site. The combined computational and functional approach used here could be applied to any other, experimental or natural channel mutations, to extract information on underlying mechanisms.

## Data Availability Statement

All datasets generated for this study are included in the article/Supplementary Material.

## Ethics Statement

The animal study was reviewed and approved by Service vétérinaire du canton de Vaud.

## Author Contributions

SV, IG, and NA carried out the experiments. OB carried out the computational work. SV, OB, and SK wrote the manuscript. All authors designed together the project.

## Conflict of Interest

The authors declare that the research was conducted in the absence of any commercial or financial relationships that could be construed as a potential conflict of interest.

## Supplementary Material

DATA SHEET S1 Tables S1–S3; Figures S1–S3.

DATA SHEET S2 Raw Data.

## Abbreviations

ASIC: Acid-Sensing Ion Channel
cASIC1: chicken ASIC
COM: center of mass
hASIC1a: human ASIC1a
MD: Molecular dynamics
pH_50_: pH of half-maximal activation
pHD_50_: pH of half-maximal desensitization
RMSD: root-mean-square deviation
SSD: Steady-state desensitization
WT: wild-type.
