# Supplementary material for: Structural and Functional Analysis of Gly212 Mutants Reveals the Importance of Intersubunit Interactions in ASIC1a Channel Function
Source: Front Mol Biosci. 2020 Apr 28;7:58. doi: 10.3389/fmolb.2020.00058 (PMC7198790; doi:10.3389/fmolb.2020.00058)
Supplement: DATA SHEET S1 — Tables S1–S3; Figures S1–S3. [file Data_Sheet_1.PDF]

Supplementary Material

Structural and functional analysis of Gly212 mutants reveals the importance of intersubunit interactions in ASIC1a channel function

Olivier Bignucolo, Sabrina Vullo, Nicolas Ambrosio, Ivan Gautschi and Stephan Kellenberger

Contents

Supplementary tables

Table S1 Molecular systems used for MD simulations

Table S2 Mutants and trajectory lengths

Table S3 Residue pairs near position 212 involved in correlated movements in the open-desensitized transition

Supplementary figures

Figure S1 Sequence alignment of ASIC1a showing the complete human ASIC1a sequence, the chicken ASIC1a of the experimentally solved structure and the modelled human ASIC1a.

Figure S2 Characterization of 212 mutants in chicken ASIC1a

Figure S3 Several side-chains near the residue 212 orient differently as a function of the mutations at position 212

Table S1. Molecular systems used for MD simulations

Protein[1]	Length[2] (# residues/ protomer)	Protein total charge[3]	Membrane composition			Water (#)	Na^+(#)	Cl^- (#)	Ca^{2+} (#)
			Lipid	Leaflet	#				
Closed	419	0	POPC	Out	123	30100	80	98	9
				In	121				
Open	514	-24	POPC	Out	140	86800	365	239	0
				In	115				
			DMPE	Out	15				
				In	15				
			DMPG	Out	52				
				In	40				
			DOPS	Out	0				
				In	10				
Des.	439	-54	POPC	Out	140	66900	339	183	0
				In	115				
			DMPE	Out	15				
				In	15				
			DMPG	Out	52				
				In	40				
			DOPS	Out	0				
				In	10				

[1]) Protein hASIC1a model, generated from the chicken open (4NTW), closed (5WKU), and desensitized (4NYK, "Des."), crystal structures.
[2]) Distinct number of residues were modelled at the N and C termini of the protein.
[3]) The calculated charge corresponds to the WT. Mutated residues were kept in their standard protonation state and the number of chloride ions was adapted to maintain an electrically neutral system.

Table S2. Mutants and trajectory lengths

Protein	Residue at position 212	Replicate #	Length (ns)	MD package
Closed	G	1	1019	GROMACS
		2	200	GROMACS
		3	209	GROMACS
	E	1	200	GROMACS
		2	200	GROMACS
		3	200	GROMACS
		4	200	GROMACS
	D	1	200	GROMACS
		2	200	GROMACS
		3	200	GROMACS
	F	1	204	GROMACS
		2	200	GROMACS
		3	200	GROMACS
Open	G	1	200	GROMACS
	E	1	200	GROMACS
	D	1	200	GROMACS
	F	1	200	GROMACS
	T	1	200	GROMACS
Des.	G	1	200	GROMACS
		2	72	NAMD
	E	1	200	GROMACS
		2	72	NAMD
	D	1	200	GROMACS
		2	72	NAMD
	F	1	200	GROMACS
	T	1	200	GROMACS

Table S3. Residue pairs near position 212 involved in correlated movements in the open-desensitized transition

Res1	Res2	Occurrences		
		Glu	Gly	Asp
363	307	6	4	3
411	274	6	6	6
311	308	5	3	5
311	307	5	3	4
409	219	4	5	2
411	217	3		
217	214	2		
216	213	2		
216	211	2		
416	82	2		
410	217		4	4
209	85		3	
216	212		2	
357	211		2	
416	277		1	
261	257			4
410	274			3
412	275			2
260	257			2

For each construct, thus G212E, G212 or G212D, the 10 most frequently observed pairs are listed. The highest possible occurrence per construct for each pair is 10. The first five lines report residue pairs that were highly correlated in all three constructs, and represent common clusters of interacting residues. Lines 6-19 report residue pairs that were unique to one construct, except for the pair 410-217 that occurred in G212 and G212D.

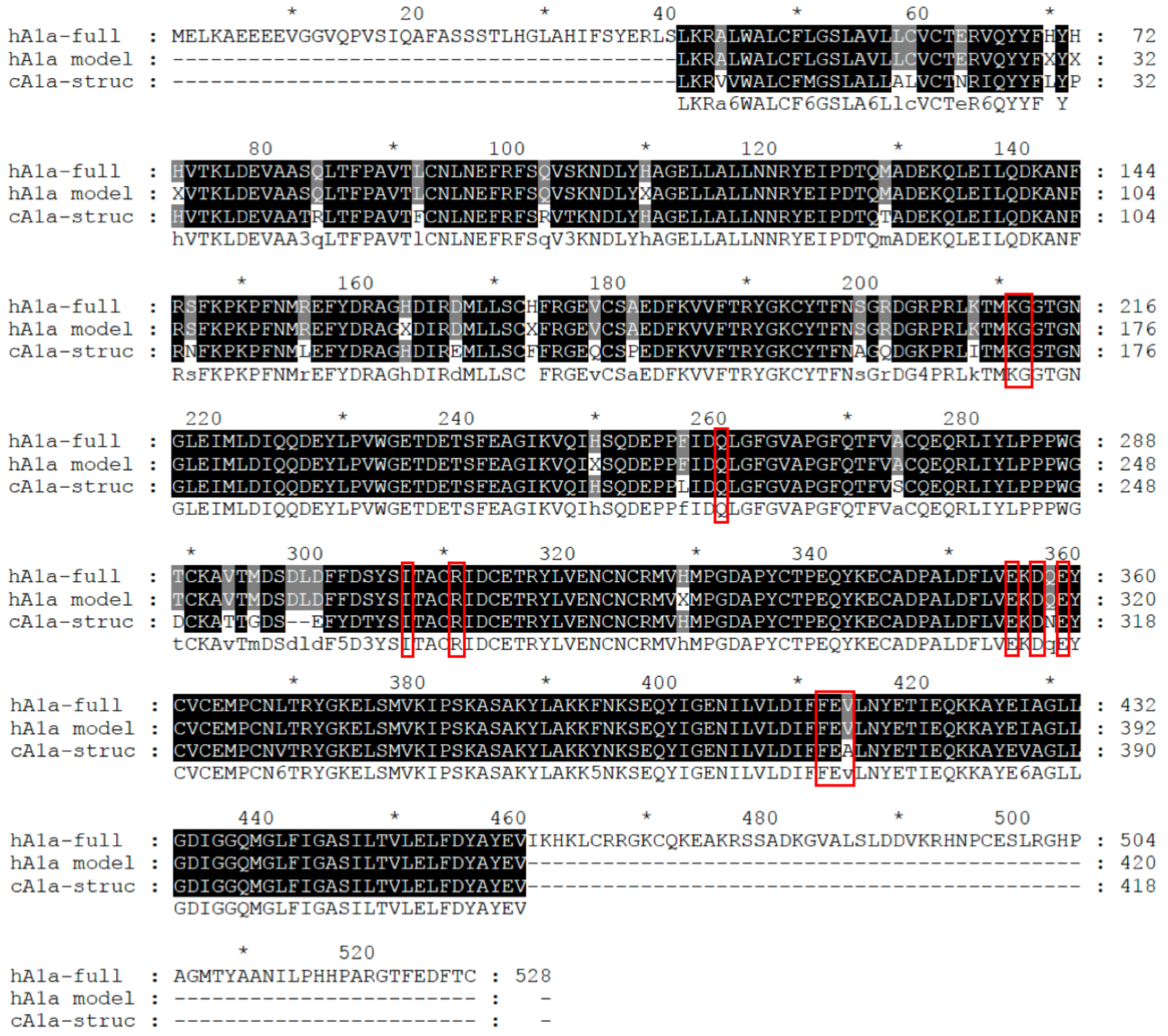

Figure S1. Sequence alignment of ASIC1a showing the complete hASIC1a sequence, the cASIC1 of the experimentally solved structure and the modelled hASIC1a. The whole sequence corresponds to the UNIPROT entry P78348, canonical sequence. hA1a-full, complete human ASIC1a sequence; hA1a-model, human ASIC1a sequence corresponding to the model of the closed conformation; cA1a-struc, chicken ASIC1a sequence corresponding to the part resolved in the closed crystal structure. Residues discussed in the text are highlighted. Note that the chicken and human sequences are identical among these residues, except for Val414, which is an Alanine in the chicken ASIC1.

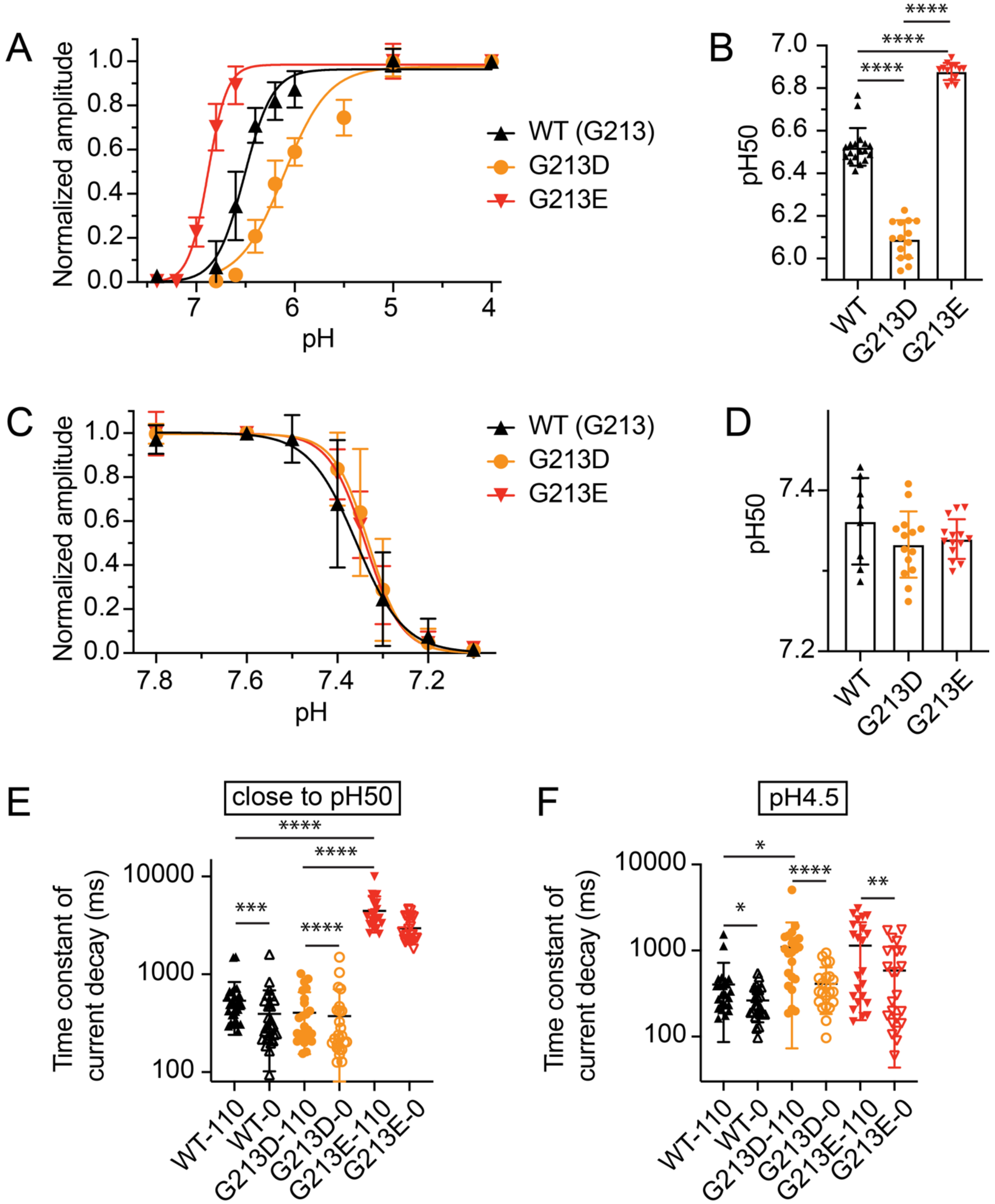

Figure S2. Characterization of 212 mutants in chicken ASIC1a. (A) activation curve of WT and Gly213 mutants (n=13-23). Between application of acidic pH, oocytes were kept at a conditioning pH of 7.8 for 50s. **(B)** plot of the pH_{50} values (n=13-18); ****, p<0.0001, ANOVA followed by Tukey post test. **(C)** SSD curve, plotting the normalized current as a function of the conditioning pH (n=8-

14). The curve was obtained by applying the conditioning pH for 55s followed by application of pH5 for 5 seconds. **(D)** plot of pHD_{50} values (n=8-14). **(E-F)** Time constant of current decay, determined at a pH value close to the pH_{50} (**E**) or at pH4.5 (**F**), in the normal recording solution containing 110mM Cl^- ("110") and in a solution in which the Cl^- was replaced by SCN^- ("0"). These values were obtained from single exponential fits to the decay phase of the current (n=22-29). The response to solutions with and without Cl^- was measured in the same oocyte. Paired t-tests were used to analyze the significance of differences between Cl^--containing and Cl^--free conditions for each mutant, and Kruskal-Wallis followed by Dunn's post test were used to analyze the significance of differences between mutants. *, $p<0.05$, **, $p<0.01$, ***, $p<0.001$ and ****, $p<0.0001$.

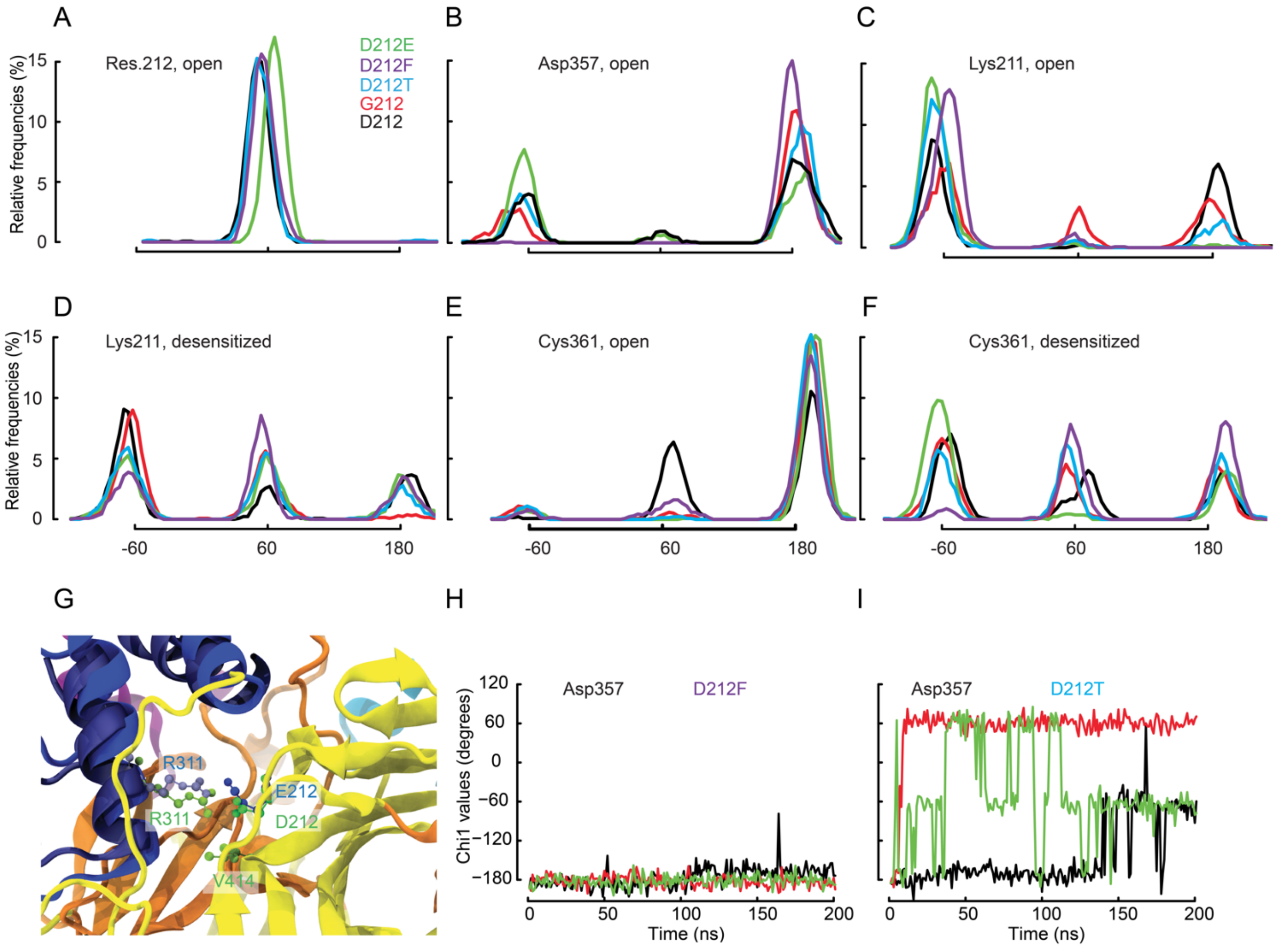

Figure S3. Several side-chains near the residue 212 orient differently as a function of the mutations at position 212. Probability densities of χ1 orientations of residues 212 (**A**) and Lys211 (**B-C**), Asp357 (**D**) and Cys361 (**E-F**) from the last 150 ns of MD trajectories. Simulations were started from the desensitized (**D, F**) or open structure (**A, B, C, E**). (**G)** Superposed molecular representations of G212D and G212E showing the heavy atoms of residues 212 for the orientation of the side chain of residue 212 in G212D where it adopted a χ1 angle of ~60°C. Selected residues interacting with residue 212 are shown in sticks. The ASIC domains are colored as follows: palm: yellow, thumb: blue, beta ball: orange, knuckle: cyan. H, I: Representative time series of Asp357 χ1 orientations in G212F (**H**) and G212T (**I**), where the trajectory of each subunit is colored differently.
